# Oral anticoagulant periprocedural management in patients undergoing an oral, dental implant or periodontal surgery: a prospective national observational survey

**DOI:** 10.1016/j.rpth.2025.102848

**Published:** 2025-04-10

**Authors:** Isabelle Mahé, David Hajage, Virginie Monnet–Corti, Société Française de Parodontologie et d’Implantologie, Louis Maman, Société Française de Chirurgie Orale, Vianney Descroix, Yann De Rycke, Loredana Radoï, PRatiques Anticoagulants oraux DIrects Chirurgie Orale group, Thong Nguyen, Roch Pecorari, Adeline Loing, Jonathan Ravasco, Tanguy Rouxel, Marion Renoux, Anne-Laure Ejeil, Nathan MOREAU, Marjolaine Gosset, Nicolas Roche, Ihsène Taïhi-Nassif, Nadia Benlagha, Juliette Rochefort, Rafael Toledo Arenas, Géraldine Lescaille, Marc Baranes, Anne-Cécile Becmeur, Sylvie Boisramé, Romain Lan, Jean-Hugues Catherine, Ugo Ordioni, Fabrice Campana, Anne-Gaëlle Chaux, Sylvian Catros, Jean-Marie Marteau, Johan Samot, Mathilde Fenélon, Julie Guillet, Charlene Kichenbrand, Bérengère Phulpin, Nicolas Glock, Cécile Chatel, Michel Guyot, François Allain, Julie Bemer, Alp Alantar, Catherine Pesci-Bardon, Oulimata Diatta-Tricon, Tariq Kabli, Moulay Chemlal

**Affiliations:** 1 Paris Cité University, AP-HP, Hôpital Louis Mourier, Service de Médecine Interne, Colombes, France; 2 Team Endotheliopathy and Hemostasis Disorders, INSERM UMR-S970, Paris Cardiovascular Research Center, Paris, France; 3 Sorbonne Université, INSERM, Institut Pierre Louis d’Epidémiologie et de Santé Publique, Paris, France; 4 Département de Santé Publique, Centre de Pharmacoépidémiologie (Cephepi), Unité de Recherche Clinique PSL-CFX, AP-HP, Hôpital Pitié Salpêtrière, Paris, France; 5 Faculté des Sciences Médicales et Paramédicales, École de Médecine Dentaire, Aix-Marseille Univ, Marseille, France; 6 AP-HM, Hôpital Timone, Pôle Odontologie, UF de Parodontologie, Marseille, France; 7 MEPHI, IRD, AP-HM, IHU Méditerranée Infection, Aix-Marseille Univ, Marseille, France; 8 on behalf of the Société Française de Parodontologie et d’Implantologie; 9 Université Paris Cité, UFR d’Odontologie, Hôpital Charles Foix, Paris, France; 10 on behalf of the Société Française de Chirurgie Orale; 11 Service Médecine Bucco–Dentaire, Institut de chirurgie dentaire–Hôpital Pitié Salpêtrière, AP-HP, LabNOF (Laboratoire de Neurobiologie OroFaciale, EA7543), Paris, France; 12 Université Paris Cité, UFR d’Odontologie, AP-HP, Hôpital Louis Mourier, Colombes, France; 13 Université Paris-Saclay, UVSQ, INSERM U1018, Gustave Roussy, CESP, Villejuif, France

**Keywords:** anticoagulant, atrial fibrillation, bleeding, oral surgery, venous thromboembolism

## Abstract

**Background:**

Oral surgery is a frequent invasive procedure in patients on oral anticoagulants. In usual care, the periprocedural management of these patients is questioned, with conflicting guidelines for patients on direct oral anticoagulants (DOACs).

**Objectives:**

To assess the risk of hemorrhagic and thromboembolic events during the periprocedural period (5 days before and 30 days after the oral invasive procedure).

**Methods:**

The PRatiques Anticoagulants oraux DIrects Chirurgie Orale Study (NCT 03150303) is a prospective noninterventional national study including patients receiving long-term oral anticoagulants (duration of treatment of at least 1 month) and referred to dental surgeons for an invasive procedure. Hemorrhagic and thromboembolic events and death on days 2, 7, and 30 were reported and analyzed according to anticoagulant management (interruption/continuation). Bleedings were classified according to the International Society on Thrombosis and Haemostasis classification after blinded review.

**Results:**

Overall, between July 2017 and December 2019, 523 patients (mean age 74) were recruited (345 on DOACs and 178 on vitamin K antagonists [VKAs]). During the periprocedural period, 62 events (11.8 events per 100 person-month [PM]) occurred, all local bleedings, in 50 patients (90.3% during the 7 days). The incidence of postoperative bleeding was greater in patients who continued on DOACs compared with patients who continued on VKAs (15.9/100 PM vs 5.9/100 PM; *P* = .003). Conversely, no significant difference was observed between patients with DOAC discontinuation and patients with VKA continuation (6.2/100 PM vs 5.9/100 PM; *P* = .75).

**Conclusion:**

In patients on anticoagulants undergoing an invasive oral procedure, the risk of bleeding occurs mainly within the 7 days following the procedure. Our data suggest the benefit of short perioperative discontinuation of DOACs.

## Essentials

• Direct oral anticoagulant (DOAC) management in patients with a dental procedure is controversial.
• This was a prospective observational study in patients on anticoagulants having a dental procedure.
• The 30-day risk of bleeding was higher in patients who continued on DOACs vs vitamin K antagonists.
• This study suggests a short DOAC discontinuation before the invasive dental procedure.

## 1 Introduction

There is a growing number of individuals prescribed anticoagulation in everyday practice as first-line treatment in patients with atrial fibrillation (AF) or mechanical prostheses for prevention of cardioembolic events and in patients with venous thromboembolic disease to prevent recurrences.

Invasive procedures are frequent in patients receiving anticoagulants, making the periprocedural management of anticoagulants a frequent situation to manage in clinical practice; it is estimated that 1 in 6 patients with AF, or an estimated 6 million patients worldwide, will require perioperative anticoagulant management [1,2]. Anticoagulant management during the perioperative period is challenging because it questions the risk of occurrence of a thrombotic event in case of anticoagulant discontinuation vs the risk of bleeding if it is continued [3].

The balance could differ according to the procedure and the type of ongoing anticoagulant that both impact the associated risk of bleeding. As a result, different options are considered in the guidelines, depending on the context [[4], [5], [6], [7]], and include anticoagulant continuation or discontinuation with or without bridging.

Oral surgery interventions are frequent invasive procedures performed in patients receiving oral anticoagulants [1]. Procedure-related bleeding risk classification based on 30-day bleeding risk [8] is not concordant, and oral surgery procedures are not singled out, most often classified as low-risk bleeding procedures (especially single tooth extraction or placement of a dental implant; Supplementary Table S1), with the possibility of managing bleeding with local hemostatic measures (compression, hemostatic agents, sutures, etc.) [3,9]. There is strong evidence from the literature in favor of continuing vitamin K antagonists (VKAs) during the oral surgery periprocedural period [[10], [11], [12], [13], [14], [15], [16], [17]], as recommended in different guidelines [6,7,18].

When direct oral anticoagulants (DOACs) were marketed, no data were available to guide prescribers in the perioperative management of treatment in terms of interruption, bridging, resumption, and/or anticoagulant level testing. From a pharmacological point of view, due to their short half-life and rapid onset of action, DOACs look attractive in the periprocedural context and offer the possibility of considering short discontinuation without requiring a bridging anticoagulant.

In practice, periprocedural DOAC management in oral surgery remains controversial due to methodological considerations and limitations of available studies [[19], [20], [21], [22]]. It remains unclear whether the same guidance as with VKA should be applied to DOACs: some of them are in favor of continuing DOACs [6,7], while others propose a brief discontinuation [4], leading to highly variable practices to date [3].

Nevertheless, several systematic reviews and meta-analyses are in favor of DOAC continuation for oral invasive dental procedures, but conclusions are based on studies at high risk of bias, majorly tooth extractions, which are not representative of the wide range of invasive procedures practiced in oral surgery, which entail different hemorrhagic risks [[23], [24], [25]]. Moreover, studies did not systematically analyze severity, time of onset, and management of postoperative bleeding, nor did they follow-up patients postoperatively for more than 7 days. Finally, conclusions are rarely supported by rigorous statistical analyses that take into account other bleeding risk factors.

Given these limitations in the literature studies on the perioperative management of patients on DOACs and VKAs undergoing oral invasive procedures, we designed a prospective observational noninterventional study with the aim to report the practices and compare the risks of 30-day bleeding and thromboembolic events (TEs) associated with the periprocedural management of anticoagulants (DOACs and VKAs) in patients undergoing any type of oral invasive procedure.

## 2 Materials and Methods

### 2.1 Study design and ethical statement

The PRatiques Anticoagulants oraux DIrects Chirurgie Orale Study was designed as a 2-stage project.

In the first step, 2 learned dental societies, the Société Française de Chirurgie Orale (SFCO) and the Société Française de Parodontologie et d’Implantologie (SFPIO), involving most French oral surgeons and periodontologists with liberal and/or hospital activity, invited their members to take part in a practice survey about the management of patients on anticoagulants in case of invasive oral procedures through mail and email. The results of this survey have been previously published [26].

In a second step, respondents were asked to participate in the PRatiques Anticoagulants oraux DIrects Chirurgie Orale Study (Oral Anticoagulant Periprocedural Management in Patients Undergoing an Oral, Dental Implant or Periodontal surgery, NCT 03150303; Appendices), a prospective national observational cohort multicenter study conducted in adult population under long-term anticoagulant treatment (DOAC or VKA) and undergoing oral surgery, implantology, or periodontology intervention by liberal and/or hospital general or specialized dentists.

A cohort design is appropriate for assessing a management strategy when expected rate of events is low and when there is sufficient statistical power to exclude clinically important higher outcome rates [27].

Patients who expressed no opposition after being informed were included in the study. The decision on the management of ongoing anticoagulant therapy was left to the discretion of the dentist, unrelated to the patient’s participation in the study. In the postprocedure setting, patients typically have a follow-up visit 7 days after the surgery with the dentist in charge of the intervention. Nevertheless, this follow-up time is insufficient to detect events associated with periprocedural management. Therefore, the follow-up duration was extended to 30 days, as recommended by the Scientific and Standardization Committee of the International Society on Thrombosis and Haemostasis (ISTH) and updated in 2019 [8,28]. The collection of follow-up information was mainly conducted by the investigators themselves during the follow-up visit on day 7, and additional follow-up information was collected through direct contact with the patients or, if appropriate, with medical staff or caregivers on day 30.

The study was sponsored by Assistance Publique des Hôpitaux de Paris (HAO-16007) and funded by SFCO, SFPIO, and an unrestricted grant from 2 companies (Bristol Myers Squibb and Daiichi Sankyo). The steering committee (Appendices) designed the study and was responsible for its conduct. The study was conducted at 25 sites in France. Practitioners were not paid for their work, and grants were dedicated to the coordination and statistics team.

The study was approved by Ile de France IV Hospital Ethics Committee (Comité de Protection des Personnes Institutional Review Board 00003835, September 29, 2016) for all French sites (N° Protocole 51NI).

A Clinical Events Committee (Appendices) unaware of the periprocedural anticoagulant management reviewed all the detailed clinical courses and adjudicated the clinical events after the completion of the study (all suspected deep vein thrombosis, pulmonary embolism, major, minor, or clinically relevant nonmajor bleeding [CRNMB] events, acute atherothrombotic cardiovascular events [ischemic stroke and acute coronary syndrome], and deaths based on results of laboratory exams and imaging or medical, medical observations, or hospitalization reports).

### 2.2 Study population

Patients aged 18 or older who were: (1). receiving long-term oral anticoagulants (defined as a duration of treatment of at least 1 month) irrespective of the indication and (2). being referred for an invasive procedure (single or multiple tooth extractions, single or multiple implant placements, bone grafting, sinus lift, mucosal/bone surgery, or other procedures at bleeding risk) to an oral surgeon or periodontologist member of the SFCO or SFPIO taking part in the study were considered for inclusion.

Oral anticoagulants of interest were as follows: VKAs: acenocoumarol, fluindione, and warfarin; DOACs: dabigatran, rivaroxaban, apixaban, and edoxaban.

Certain eligibility criteria were changed during the study. Initially, patients receiving a concomitant antiplatelet agent were excluded until May 2019. In order to also assess the frequent situation of patients receiving concomitant anticoagulants and antiplatelet agents, those patients were authorized to participate in the study from May 2019 to the end of the study after a protocol amendment.

### 2.3 Data collection

Baseline patient information, including demographics, medications, and comorbidities, was recorded at screening visit. Information about medication changes, new comorbidities, and bleeding or thrombotic events between screening and inclusion visits (day of surgery) were abstracted for each patient.

The following data were collected in the periprocedural period:

- Preoperative data included the date of procedure, anticoagulation management before the procedure (if so, interruption, switch, and restart dates, and whether or not heparin bridging was used), and the type of procedure.
- Postoperative data: all patients underwent a follow-up visit at 7 days and were contacted at 30 days. At each contact, patients were asked about the occurrence of hemorrhagic events (within the 24 postoperative hours, and from day 1 to day 7), qualitative appreciation of the bleeding, management of the bleeding, recommendations for the anticoagulant treatment, TEs or other local or systemic complications, visits to the hospital or a physician, changes in medications, and diagnostic tests undertaken because of complications, if any. In case of a suspected outcome event, an adjudication package containing all relevant information was submitted for adjudication by a specific ad hoc committee (Appendices).

### 2.4 Outcomes

The primary outcome of the study was the composite rate of 30-day TEs (including transient ischemic attacks, ischemic strokes [29], noncerebrovascular systemic embolism [29], and recurrent venous thromboembolism [30]) and bleeding (including major bleeding, CRNMB, and minor bleeding) occurring in the periprocedural period in patients on long-term DOACs and VKAs and undergoing an oral invasive procedure [31] (Supplementary Material). Criteria for major bleedings and CRNMBs were based on the ISTH definitions [32,33] (Supplementary Material).

The periprocedural period was defined as the period between 5 days before surgery and 30 days postprocedure (if no anticoagulant discontinuation) or 30 days after the resumption of oral anticoagulants (in case of an anticoagulant discontinuation) [28].

Secondary endpoints comprised the bleeds, TEs, and risk factors associated with these events. Periprocedural anticoagulant continuation was considered for 1/patient continuing the usual anticoagulant at the same dose, 2/patients on VKAs with low-molecular-weight heparin (LMWH) bridging, 3/patients on VKAs with temporary dosage lowering, and 4/patients on DOACs who switched to LMWH. Periprocedural anticoagulant discontinuation was defined as VKA withdrawal 3 days or more before the procedure, without any bridging, or DOAC withdrawal at least the day before the procedure (last medication taken no later than the morning before the procedure) [6].

The procedures were categorized into having low or high bleed risk based on categories from the SFCO classification (Supplementary Table S1) [6].

### 2.5 Sample size

We estimated that at least 60 practitioners, members of the SFCO, and at least 40 practitioner members of the SFPIO would agree to participate, and each practitioner would include 10 patients receiving long-term VKAs and 10 patients receiving long-term DOACs. Overall, we planned to include 1000 patients in each group (VKAs and DOACs) and 2000 patients overall.

After a dental avulsion, the risk of postoperative bleeding varies between 2% and 7% [15,34,35], depending on the study, in case of VKA continuation. The risk of a TE is lower in case of VKA continuation, between 0.5% and 1% [36].

The primary objective of the study was to estimate the incidence rate of TEs and bleeding events in the periprocedural period in the VKA and DOAC groups. Assuming a 5% incidence rate of TEs and bleeding events in the periprocedural period (primary outcome), the inclusion of 1000 patients in each group would allow a precision (half-width of the 95% CI) of 1.4% for this estimation.

### 2.6 Statistical analysis

Characteristics of patients and anticoagulant management were described with frequencies and percentages for categorical variables and mean ± SD for continuous variables, as appropriate. Unless otherwise specified, categorical variables were compared with chi-squared tests or Fisher’s exact tests, and continuous variables were compared with Student’s *t*-test or Wilcoxon test, as appropriate.

The proportion of each type of event in each group was estimated with its exact binomial 95% CI. Incidence rates of events per person-month (PM) were also provided, with 95% CIs estimated using a Poisson model with the length of follow-up as an offset term (the addition of an offset term is a modeling technique in Poisson regression that allows for modeling rates instead of counts). Since the exact date of events that occurred in the periprocedural period was not available, the event time was imputed to the end of the follow-up interval. The comparisons of proportions were performed using chi-squared tests or Fisher exact tests, and the comparisons of incidence rates using a Poisson model.

Risk factors (age, gender, indication for anticoagulation, class of anticoagulant, preprocedure discontinuation of anticoagulant, if any, time between the last intake of the anticoagulant treatment and the procedure, bleeding risk of the procedure, and comorbidities) associated with hemorrhagic events and TEs were analyzed in 2 steps. First, a univariable analysis was performed, using Student’s *t*-test or Wilcoxon test for continuous risk factors and chi-squared tests or Fisher’s exact tests for categorical risk factors. Factors associated (*P* < .10) with outcome in the univariable analysis were introduced into a multivariable logistic model to estimate odds ratios and 95% CIs for occurrence events vs no occurrence. Since no TEs were observed in the study, this analysis was performed on bleeding events only. An analysis limited to CRNMB was also performed using the same variables.

We used a complete cases approach for all analyses (no imputations). All analyses were conducted at 2-sided α risk of 5% by an academic biostatistician using the R software version 4.1 (R Foundation for Statistical Computing).

## 3 Results

The recruitment was stopped prematurely for funding reasons on December 9, 2019, after 523 patients were included.

### 3.1 Study population and patient characteristics

**Figure 1:**
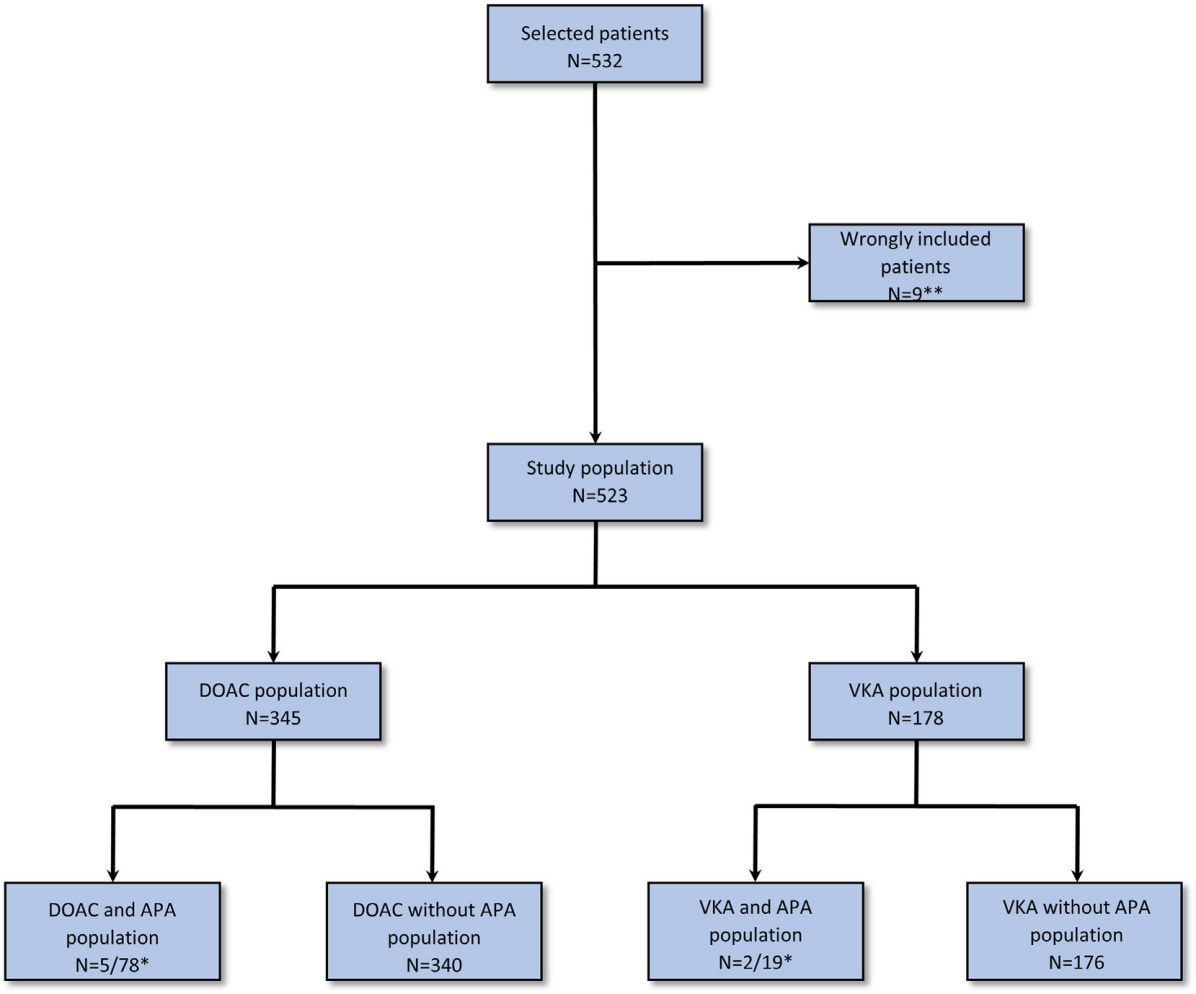
Flow chart. ∗For patients included before May 27, 2019, none was receiving antiplatelet treatment (exclusion criteria). ∗∗Five patients did not undergo invasive procedures, 2 patients were not on long-term treatment with an oral anticoagulant, 1 patient was treated with both a direct oral anticoagulant (DOAC) and an antiplatelet agent, and 1 patient was treated with both a vitamin K antagonist (VKA) and an antiplatelet agent. APA, antiplatelet agents.

From July 2017 to December 2019, 178 (34%) VKA-treated and 345 (66%) DOAC-treated patients met inclusion/exclusion criteria for the study and were included (Figure 1). All patients except 5 were followed up at 30 days. Two patients died with no hemorrhagic event or TE (1 episode of acute respiratory failure and 1 cardiorespiratory decompensation due to chronic obstructive pulmonary disease secondary infection). Three other patients were lost to follow-up between day 7 and day 30, with no hemorrhagic events or TEs before day 7. To avoid overestimating the incidence of events, these patients were considered as having no event during the follow-up period.

At the time of inclusion, the characteristics of the 2 treatment groups (VKA, DOAC) were well-balanced (Table 1). The mean (SD) age of participants was 73.8 ± 12.7 years; more than half of the patients (58.1%) had cardiovascular disease, and about 20% had diabetes. In the DOAC group, 5 patients also received antiplatelet therapy (3 aspirin and 2 clopidogrel) vs 2 patients in the VKA group (1 aspirin and 1 clopidogrel).

**Table 1:** Characteristics of patients and anticoagulant management according to ongoing anticoagulant before procedure.

Variable	Apixaban (*n* = 123) *n* (%)	Rivaroxaban (*n* = 186) *n* (%)	Dabigatran (*n* = 36) *n* (%)	DOAC (*n* = 345) *n* (%)	VKA (*n* = 178) *n* (%)	Total (*N* = 523) *n* (%)	*P* value^b^
Gender, male	58 (47.2)	113 (60.8)	27 (75)	198 (57.4)	93 (52.2)	291 (55.6)	.262
Age (y), mean ± SD	76.06 ± 11.52	73.03 ± 11.73	72.39 ± 11.16	74.04 ± 11.66	73.35 ± 14.42	73.80 ± 12.66	.58
Indication for anticoagulation^c^							
Atrial fibrillation	92 (74.8)	136 (73.1)	32 (88.9)	260 (75.4)	92 (51.7)	352 (67.3)	<.0001
DVT and/or PE	22 (17.9)	33 (17.7)	4 (11.1)	59 (17.1)	39 (21.9)	98 (18.7)	.182
Valve prosthesis^c^	4 (3.3)	7 (3.8)	0 (0)	11 (3.2)	49 (27.5)	60 (11.5)	<.0001
Other^d^	12 (9.8)	18 (9.7)	1 (2.8)	31 (9)	12 (6.7)	43 (8.2)	.376
History of comorbidities^c^^,^^f^							
Cardiovascular disease	75 (61)	115 (61.8)	23 (63.9)	213 (61.7)	91 (51.1)	304 (58.1)	.0197
Pulmonary	18 (14.6)	18 (9.7)	0 (0)	36 (10.4)	19 (10.7)	55 (10.5)	.933
Hematological	6 (4.9)	8 (4.3)	0 (0)	14 (4.1)	8 (4.5)	22 (4.2)	.814
Infectious	1 (0.8)	2 (1.1)	1 (2.8)	4 (1.2)	1 (0.6)	5 (1)	.666
Diabetes	17 (13.8)	39 (21)	13 (36.1)	69 (20)	35 (19.7)	104 (19.9)	.927
Hepatic/gastrointestinal	7 (5.7)	15 (8.1)	2 (5.6)	24 (7)	8 (4.5)	32 (6.1)	.266
Stroke	26 (21.1)	15 (8.1)	4 (11.1)	45 (13)	20 (11.2)	65 (12.4)	.553
Renal impairment	6 (4.9)	9 (4.8)	0 (0)	15 (4.3)	17 (9.6)	32 (6.1)	.019
Cancer	13 (10.6)	25 (13.4)	6 (16.7)	44 (12.8)	11 (6.2)	55 (10.5)	.020
Procedures							
Single tooth extraction	47 (38.2)	81 (43.5)	17 (47.2)	145 (42)	67 (37.6)	212 (40.5)	.333
Multiple teeth extractions in the same quadrant	34 (27.6)	38 (20.4)	7 (19.4)	79 (22.9)	46 (25.8)	125 (23.9)	.454
Multiple teeth extractions in different quadrants	36 (29.3)	54 (29)	11 (30.6)	101 (29.3)	55 (30.9)	156 (29.8)	.701
Others^e^	9 (7.3)	16 (8.6)	1 (2.8)	26 (7.5)	15 (8.4)	41 (7.8)	.7195
Anticoagulation before procedure^a^							.089
Discontinuation without bridging	10 (8.1)	20 (10.8)	2 (5.6)	32 (9.3)	9 (5.1)	41 (7.8)	
Continuation	113 (91.9)	166 (89.2)	34 (94.4)	313 (90.7)	169 (94.9)	482 (92.2)	
Ongoing anticoagulant in patients with anticoagulant stop without bridging							
d 7	10 (100)	20 (100)	2 (100)	32 (100)	9 (100)	41 (100)	1.00
d 30	10 (100)	17 (85)	2 (100)	29 (90.6)	9 (100)	38 (92.7)	1.00

DOAC, direct oral anticoagulant; DVT, deep vein thrombosis; PE, pulmonary embolism**;** VKA, vitamin K antagonist.

a After adjudication.

b *P* values of tests comparing VKA and DOAC groups.

c As reported by patients to dentists.

d Other: coronary artery bypass, aortic aneurysm, history of stroke, catheter vein thrombosis, dilated cardiomyopathy, ischemic cardiomyopathy, Vaquez disease, myeloma, ventricular tachycardia, and aortic/mitral insufficiency.

e Other procedures: dental implant placement or removal, preimplant surgery, preprosthetic surgery, periodontal/mucosal surgery, biopsy (bone/mucosa), and periodontal pocket surgery.

f Two missing comorbidity data.

In terms of indication, a higher proportion of patients with AF were receiving DOACs (75.4% vs 51.7%, *P* < .0001), and a higher proportion of patients with valve prostheses were receiving VKAs (27.5% vs 3.2%, *P* < .0001).

Before the procedure, anticoagulant treatment was discontinued in 41 patients (7.8%; Table 1). For patients with preprocedure anticoagulant discontinuation (without bridging/switching), the median delay from anticoagulant discontinuation to the procedure was 2.00 (2.00; 2.00) days for the DOAC group and 3.00 (2.00; 4.00) days for the VKA group, *P* = .008. Anticoagulant treatment was continued in 482 patients (92.2%; 313 patients [91%] on DOACs and 169 patients [95%] on VKAs, *P* > .05); 460 continued the usual anticoagulant at the same dose (306 patients on DOACs and 154 patients on VKAs), and 22 patients had anticoagulant continuation (7 patients on DOACs and 15 patients on VKAs) as follows: 6 patients on VKAs underwent LMWH bridging, 5 patients on DOACs were switched to LMWH and 9 patients to VKAs (with an international normalized ratio [INR] ranging from 1.99 to 3.6 prior to the procedure), and 2 patients on DOACs had temporary dosage lowering.

**Table 2:** Characteristics of patients experiencing bleeding vs patients not experiencing bleeding during the periprocedural period.

Variable	No periprocedural bleeding (*n* = 473) *n* (%)	Periprocedural bleeding (*n* = 50) *n* (%)	Total (*N* = 523) *n* (%)	*P* value
Gender, male	255 (53.9)	36 (72)	291 (55.6)	.01
Age (y), mean ± SD	73.67 ± 12.85	75.08 ± 10.75	73.80 ± 12.66	.76
Indication for anticoagulation^a^				
Atrial fibrillation	293 (62.9)	32 (64.0)	325 (62.1)	.45
DVT and/or PE	89 (18.8)	8 (16.0)	97 (18.5)	
Valve prosthesis^a^	59 (12.5)	4 (8.0)	63 (12.0)	
Other^b^	32 (6.8)	6 (12.0)	38 (7.3)	
History of comorbidities^a^^,^^c^				
Cardiovascular disease	273 (58.0)	31 (62.0)	304 (58.3)	.58
Pulmonary disease	49 (10.4)	6 (12.0)	55 (10.6)	.72
Hematological disease	17 (3.6)	5 (10.0)	22 (4.2)	.04
Infectious disease	5 (1.1)	0 (0)	5 (1.0)	1.00
Hepatic/gastrointestinal disease	27 (15.7)	5 (10.0)	32 (6.1)	.37
Diabetes	91 (19.3)	13 (26.0)	104 (20.0)	.26
Stroke	59 (12.5)	6 (12.0)	65 (12.5)	.91
Renal impairment	30 (6.4)	2 (4.0)	32 (6.1)	.72
Cancer	49 (10.4)	6 (12.0)	55 (10.6)	.72
Dental procedure-related bleeding risk classification				
Low risk	316 (66.8)	27 (54.0)	343 (65.6)	.06
High risk	157 (33.2)	23 (46.0)	180 (34.4)	
Preprocedure type of anticoagulant and its management				
VKA	170 (35.9)	8 (16.0)	178 (34.0)	.02
Discontinuation	9 (5.3)	0 (0.0)	9 (5.1)	
Continuation	161 (94.7)	8 (100.0)	169 (94.9)	
DOAC	303 (64.1)	42 (84.0)	345 (66.0)	
Discontinuation	30 (9.9)	2 (4.8)	32 (9.3)	
Continuation	273 (90.1)	40 (95.2)	313 (90.7)	

DOAC, direct oral anticoagulant; DVT, deep vein thrombosis; PE, pulmonary embolism; VKA, vitamin K antagonist.

a As reported by patients to dentists.

b Other: coronary artery bypass, aortic aneurysm, history of stroke, catheter vein thrombosis, dilated cardiomyopathy, ischemic cardiomyopathy, Vaquez disease, myeloma, ventricular tachycardia, and aortic/mitral insufficiency.

c Two missing comorbidity data.

Antiplatelet treatment was continued during the perioperative period.

Procedures with different bleeding risks were represented in the study: single tooth extraction (42% in DOAC group and 37.6% in the VKA group, *P* > .05), multiple teeth extractions in the same quadrant (22.9% of patients in DOAC group and 25.8% in VKA group, *P* > .05), and multiple teeth extractions in different quadrants (29.3% in the DOAC group and 30.9% in the VKA group, *P* > .05).

### 3.2 Outcomes

#### 3.2.1 Hemorrhagic events and TEs

No TEs were recorded during the periprocedural period. Thus, the remainder of this section focuses on hemorrhagic events only.

#### 3.2.2 Bleedings

**Figure 2:**
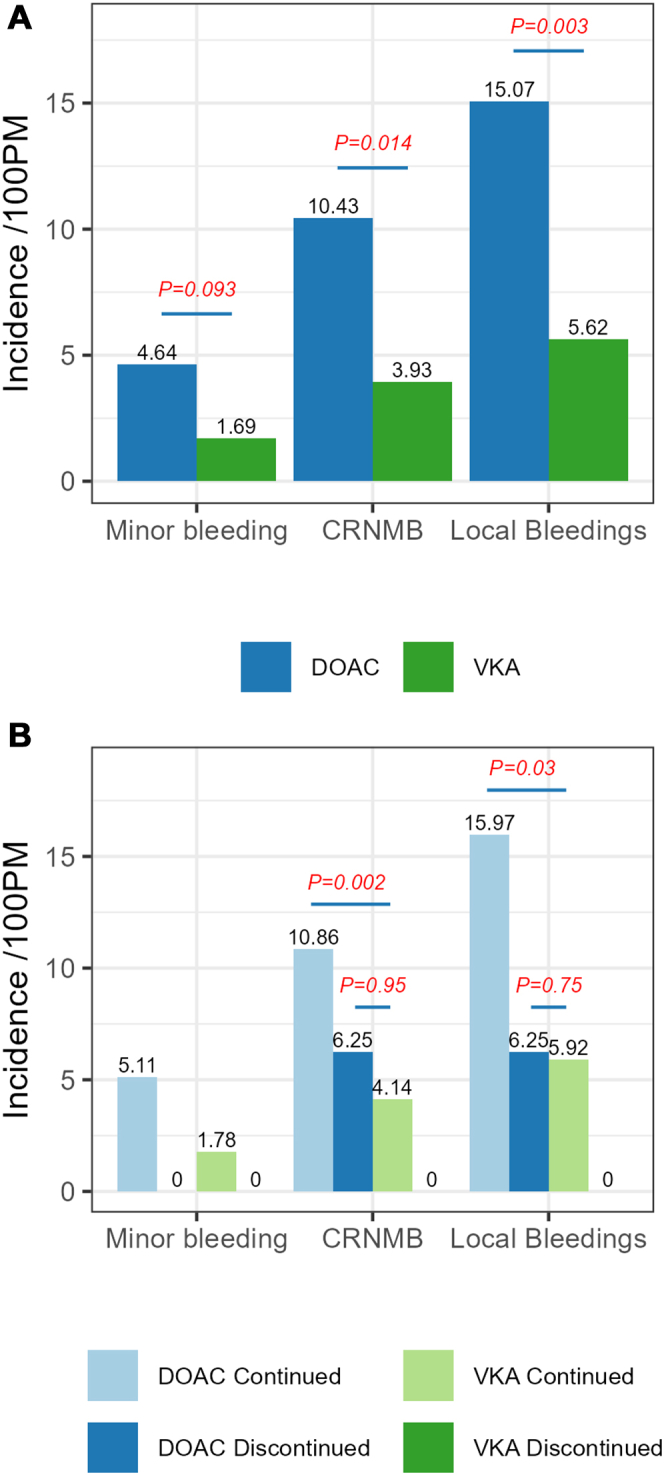
(A) Total, minor, and clinically relevant nonmajor bleeding (CRNMB) incidence rates by anticoagulant. (B) Total, minor, and CRNMB incidence rates by anticoagulant and preprocedural management. DOAC, direct oral anticoagulant; PM, person-month; VKA, vitamin K antagonist.

During the periprocedural period of 30 days, 62 bleedings occurred in 50 patients (Figure 2, Supplementary Table S2). The global incidence of bleeding was 11.85 (95% CI [9.24, 15.21]) events per 100 PM. The total percentage of subjects with at least 1 bleeding within 30 days was 9.56% (7.18%, 12.41%).

Of the 62 bleedings, 43 (69.4%) CRNMBs and 19 (30.6%) minor bleedings were recorded. No major bleeding was reported (Supplementary Table S2). All bleedings meeting the criteria of CRNMB were bleedings associated with unscheduled contact with a physician (visit) and medical intervention.

The global incidence rate of CRNMB was 8.22 (95% CI [6.10, 11.09]) events per 100 PM. The total percentage of subjects with at least 1 CRNMB within 30 days was 6.88% (4.87%, 9.40%).

The global incidence rate of minor bleeding was 3.63 (95% CI [2.32, 5.70]) events per 100 PM. The total percentage of subjects with at least 1 minor bleeding within 30 days was 3.63% (2.20%, 5.62%). Around one-third of the hemorrhagic events occurred within 24 hours (30.6%) and less than two-third (59.7%) between day 2 and day 7. Less than 10% (9.7%) occurred between day 7 and day 30 (Supplementary Table S2).

#### 3.2.3 Sites of bleeding

In the entire study population, all bleedings were local bleedings, which were stopped by the dentists using local hemostasis methods.

#### 3.2.4 Bleeding and type of anticoagulant

The global incidence rate of bleeding was 15.07 (95% CI [11.49, 19.78]) events per 100 PM in patients on DOACs and lower at 5.62 (95% CI [3.02, 10.44]) events per 100 PM in patients on VKAs (*P* = .003; Figure 2A, Supplementary Table S2).

The total percentage of subjects with at least 1 bleeding within 30 days was higher in the DOAC group than in the VKA group: 12.17% (8.92%, 16.10%) vs 4.49% (1.96%, 8.66%; *P* = .005; Supplementary Table S2).

The global incidence rate of bleeding was higher in the DOAC group compared with VKA group for both minor bleeding (4.64 [2.84, 7.57] vs 1.69 [0.54, 5.23] events per 100 PM, *P* = .09) and CRNMB (10.43 [7.53, 14.47] vs 3.93 [1.87, 8.25] events per 100 PM, *P* = .01; Figure 2A, Supplementary Table S2).

#### 3.2.5 Bleeding according to anticoagulation therapy continuation or discontinuation

##### 3.2.5.1 Continuation

The global incidence rate of bleeding in patients with DOAC continuation was 15.97 (95% CI [12.11, 21.08]) events per 100 PM, higher than the global incidence rate of bleeding in patients who continued on VKAs: 5.92 (95% CI [3.18, 11.00]) events per 100 PM (*P* = .003; Supplementary Table S2, Figure 2B).

The total percentage of subjects with at least 1 bleeding within 30 days was higher in the uninterrupted DOAC group than in the uninterrupted VKA group: 12.78% (9.29%, 16.99%) vs 4.73% (2.07%, 9.11%), *P* = .005 (Supplementary Table S2).

The incidence rate of bleeding was higher in the uninterrupted DOAC group compared with uninterrupted VKA group for both minor bleeding (5.11 [3.13, 8.34] vs 1.78 [0.57, 5.50] events per 100 PM, *P* = .078) and CRNMB (10.86 [7.76, 15.20] vs 4.14 [1.97, 8.69] events per 100 PM, *P* = .016; Supplementary Table S2, Figure 2B).

##### 3.2.5.2 Discontinuation

The global incidence rate of bleeding in patients on interrupted DOACs was 6.25 (95% CI [1.56, 24.99]) events per 100 PM, and no event was observed in the group of interrupted VKAs (Supplementary Table S2, Figure 2B).

The total number of subjects with at least 1 bleeding within 30 days was low (2 patients in the interrupted DOAC group and 0 patients in the interrupted VKA group; Supplementary Table S2).

The incidence rate of CRNMB was higher in the interrupted DOAC group compared with interrupted VKA group (6.25 [1.56, 24.99] vs 0 events per 100 PM, *P* = .917). No minor bleedings were recorded in both discontinued treatment groups (Supplementary Table S2, Figure 2B).

#### 3.2.6 Comparison of DOAC discontinuation vs VKA continuation

Comparison of groups according to preoperative continuation or interruption of the anticoagulation showed that occurrence of overall and CRNMB postoperative bleedings was similar in patients with DOAC discontinuation compared with patients who continued VKAs, with 6.25 (1.56, 24.99) events per 100 PM vs 5.92 (3.18, 11.00) events per 100 PM, *P* = .746, respectively, and 6.25 (0.77, 20.21) events vs 4.14 (1.97, 8.69) events per 100 PM, *P* = .95, respectively, (Supplementary Table S2, Figure 2B).

#### 3.2.7 Bleeding vs no bleeding (total and CRNMB)

Characteristics of patients did not significantly differ across patients experiencing bleeding vs those not experiencing bleeding during the periprocedural period with respect to age, indications of anticoagulation, comorbidities, and the surgical procedure. Patients with bleeding were more frequently men (*P* = .01; Tables 2 and 3). No difference in the proportion of dental procedures at high risk of bleeding between men and women was observed (*P* = .85).

**Table 3:** Characteristics of patients experiencing clinically relevant nonmajor bleedings vs patients not experiencing clinically relevant nonmajor bleedings during the periprocedural period.

Variables	No periprocedural bleeding (*n* = 473) *n* (%)	Periprocedural bleeding (*n* = 36) *n* (%)	Total (*N* = 509) *n* (%)	*P* value
Gender, male	255 (53.9)	27 (75.0)	282 (55.4)	.01
Age (y), mean ± SD	73.67 ± 12.85	75.81 ± 11.06	73.82 ± 12.73	.51
Indications for anticoagulation^a^				
Atrial fibrillation	293 (61.9)	20 (55.6)	313 (61.5)	.27
DVT and/or PE	89 (18.8)	7 (19.4)	96 (18.9)	
Valve prosthesis^a^	59 (12.5)	3 (8.3)	62 (12.2)	
Other^b^	32 (6.8)	6 (16.7)	38 (7.5)	
History of comorbidities^a^^,^^c^				
Cardiovascular disease	273 (58.0)	22 (61.1)	295 (58.2)	.71
Pulmonary disease	49 (10.4)	5 (13.9)	54 (10.7)	.71
Hematological disease	17 (3.6)	4 (11.1)	21 (4.1)	.05
Infectious disease	5 (1.1)	0 (0)	5 (1.0)	1.00
Diabetes	91 (19.3)	9 (25.0)	100 (19.7)	.41
Hepatic/gastrointestinal disease	27 (5.7)	4 (11.1)	31 (6.1)	.26
Stroke	59 (12.5)	4 (11.1)	63 (12.4)	1.0
Renal impairment	30 (6.4)	0 (0)	30 (5.9)	.15
Cancer	49 (10.4)	5 (13.9)	54 (10.7)	.71
Dental procedure-related bleeding risk classification				
Low	316 (66.8)	19 (52.8)	335 (65.8)	.08
High	157 (33.2)	17 (47.2)	174 (34.2)	
Preprocedure type of anticoagulant and its management				
VKA	170 (35.9)	6 (16.7)	176 (34.6)	
Discontinuation	9 (5.3)	0 (0)	9 (5.1)	.11
Continuation	161 (94.7)	6 (100.0)	167 (94.9)	
DOAC	303 (64.1)	30 (83.3)	333 (65.4)	
Discontinuation	30 (9.9)	2 (6.7)	32 (9.6)	
Continuation	273 (90.1)	28 (93.3)	301 (90.4)	

DOAC, direct oral anticoagulant; DVT, deep vein thrombosis; PE, pulmonary embolism; VKA, vitamin K antagonist.

a As reported by patients to dentists.

b Other: coronary artery bypass, aortic aneurysm, history of stroke, catheter vein thrombosis, dilated cardiomyopathy, ischemic cardiomyopathy, Vaquez disease, myeloma, ventricular tachycardia, and aortic/mitral insufficiency.

c Two missing comorbidity data.

### 3.3 Risk factors associated with bleeding in the perioperative period

Variables with a *P* value of less than .10 (gender, treatment class and its management, and the bleeding risk of interventions according to their invasiveness [classification SFCO]) were included in the multivariable analysis (Table 4).

**Table 4:** Associations between the 30-day risk of bleeding and selected risk factors.

Variables		*n*	OR^a^	95% CI	*P* value
**CRNMB**					
Gender	Male	281	1 (ref)	-	.02
	Women	226	0.41	0.19, 0.91	
Anticoagulant management	VKA	176	1 (ref)	-	.038
	DOAC continuation	299	2.89	1.17, 7.17	
	DOAC discontinuation	32	1.51	0.28, 7.99	
Dental procedure-related bleeding risk classification (SFCO classification)	Low risk	333	1 (ref)	-	.104
	High risk	174	1.79	0.89, 3.60	
**All bleedings (CRNMB and minor)**					
Gender	Male	290	1 (ref)	-	.02
	Women	231	0.48	0.25, 0.93	
Anticoagulant management	VKA	178	1 (ref)	-	.005
	DOAC continuation	311	3.13	1.43, 6.89	
	DOAC discontinuation	32	1.15	0.23, 5.80	
Dental procedure-related bleeding risk classification (SFCO classification)	Low risk	341	1 (ref)	-	.067
	High risk	180	1.77	0.97, 3.24	

CRNMB, clinically relevant nonmajor bleeding; DOAC, direct oral anticoagulant; OR, odds ratio; ref, reference; SFCO, Société Française de Chirurgie Orale (French Society of Oral Surgery); VKA, vitamin K antagonist.

a Results of the multivariate logistic regression. Two patients with missing comorbidity information were excluded from univariable and multivariable analyses.

Compared with the VKAs, the risk of global bleeding and CRNMB was respectively 3.1- and 2.9-fold higher when DOACs were continued and close to 1 when DOACs were stopped in the preoperative period. Procedures at high risk of bleeding were associated with a nonsignificant 1.8-fold increased risk of events compared with low-risk procedures (Table 4).

## 4 Discussion

We report the first prospective study dedicated to assessing the course and the risks associated with the periprocedural management of anticoagulants, including local and general complications, in patients undergoing oral invasive procedures with a 30-day follow-up. Of note, it is a frequently encountered situation as more than 10% of patients receiving oral anticoagulants require the management of treatment before an invasive procedure [27]. Both the risk of thrombotic complication in case of anticoagulant discontinuation and the risk of bleeding in case of anticoagulant continuation have to be considered and balanced when an invasive procedure is required in a patient receiving an anticoagulant. The decision must be based on the trade-off between the expected risk of thrombotic and hemorrhagic complications, which is specific to each procedure [8].

A meta-analysis reported a rate of global bleeding associated with dental surgeries (including mostly dental extractions and dental implant surgeries) in individuals not receiving oral anticoagulants of around 1% [[37], [38], [39]], lower than the global rate of bleeding in patients receiving VKAs or DOACs (4.33%) after follow-up periods of up to 14 days [39]. The main findings in the present study were as follows. First, with contemporary and current practices, all bleeding complications occurring during the periprocedural period in patients on anticoagulants undergoing oral surgery were local bleedings that could be managed by the dentist. No patient experienced major bleeding or thromboembolic complications; one-third were minor bleeds, and two-thirds were CRNMBs. Around one-third of all hemorrhagic events occurred immediately within 24 hours after the procedure, and two-thirds between days 2 and 30. When considering the CRNMB, half occurred within the first 24 hours and half between days 2 and 7. Second, in the context of invasive oral procedures, compared with patients with VKA continuation (same dosage or lowered dosage with an INR still in a therapeutic range considered a standard of care or with LMWH bridging), the risk of 30-day bleeding (total or CRNMB) was 3-fold higher when DOACs were continued, and very similar in patients with a short DOAC discontinuation before the procedure. This finding was observed at any time interval following the procedure (24 hours, day 7, and day 30).

Our results suggest that it may be preferable to interrupt DOAC treatment briefly before an invasive oral procedure, in the same way as other medical procedures at low risk of bleeding (eg, digestive or urologic procedures).

DOACs have arrived more recently, with recommendations from learned societies based on pooled perioperative data in the literature from patients undergoing a variety of procedures, without distinguishing dental procedures. On the basis of the short half-life of DOACs, for procedures with a generally low risk of bleeding, international guidelines agree on the need for a brief interruption, with a last dose of anticoagulant taken the morning before the procedure, followed by rapid resumption on the day of the procedure without any bridging [4,6,18]. However, this proposal is controversial for French dentists/oral surgeons, who prefer to continue DOAC treatment based on the recommendations of the SFCO published in 2015, which extrapolated the VKA management model to DOACs [6].

Recent studies are in favor of DOAC continuation for oral invasive procedures, but conclusions are limited by methodological considerations [[40], [41], [42], [43]]. A recent meta-analysis comparing the bleeding outcomes after dental extraction in patients under uninterrupted DOACs vs VKAs has included 8 studies comparing 539 patients on DOAC therapy and 574 patients on VKAs. It concluded, based on studies of very low quality that patients on DOACs may have a reduced risk of hemorrhage, and incited to interpret this result with caution [23]. Another meta-analysis showed that there was no significant difference in the bleeding risk between patients continuing or discontinuing VKAs while undergoing dental extractions, and also no significant difference in postoperative bleeding risk at 1 day and 7 days [25]. A systematic review of the effects of the discontinuation of anticoagulant therapy and postoperative bleeding after simple dental extraction in patients under DOACs and VKAs has shown that the most common complication was the immediate postoperative bleeding, mostly minor, in both groups, with no significant difference in the bleeding rates between the DOACs and VKAs [24], but this conclusion was not based on any quantitative analysis. However, the 7 included studies had bias, and the management of treatments and the hemostatic measures were different from one study to another, which may have influenced the bleeding rates. Moreover, published articles are majorly based on tooth extractions, which are not representative of the wide range of invasive procedures practiced in oral surgery, which entail different hemorrhagic risks.

None of the studies investigated the risk of thromboembolism, and those reporting hemorrhagic events did not systematically report the delay of bleeding onset, the severity, and management of postoperative bleeding, nor followed up with patients postoperatively for more than 7 days. However, complications can occur up to 30 days postoperatively, warranting longer follow-up of patients.

In a single-center, prospective, cohort study (Dental Extractions on NOAC Without Stopping Therapy Study), 7-day bleeding outcomes were compared between patients on continued DOACs and patients on continued warfarin with an INR between 2.0 and 4.0 [41]. A total of 195 teeth were extracted from 107 patients: 50 teeth from 21 patients on warfarin and 145 teeth from 86 patients on a DOAC (41 apixaban, 30 rivaroxaban, and 15 dabigatran). In this study, continuing DOACs at the time of teeth extractions led to bleeding rates similar to patients on warfarin (36% and 43%, respectively). The majority of the bleeding episodes were minor, with only 6% of DOAC patients developing a CRNMB in the 7 days following dental extractions vs 10% in patients on warfarin. Adjusted odds ratios comparing patients with bleeding and those without postoperative events showed comparable risks of bleeding in both groups (DOACs and VKAs), suggesting that there is no need to adjust DOAC dosing prior to dental extractions, nor is there a need to time the dental extractions around DOAC doses. In contrast to the study of Brennan et al. [41], our study found a 3-fold higher risk of bleeding in patients continued on DOACs vs those continued on VKAs and a greater number of CRNMBs (around two-thirds).

However, in patients who continued on VKAs and those with DOAC discontinuation, 5.9 and 6.2 events per 100 PM occurred within the 30 days following the procedure, respectively, more than 80% of which occurred within the 7 days following the procedure. In this way, our results are in accordance with the strategy proposed in the Perioperative Anticoagulant Use for Surgery Evaluation (PAUSE) Study [27] and Groupe d'Interêt en Hémostase Péri-opératoire.(GIHP) group [4], where the DOAC regimens were omitted the day before a procedure.

Regarding the type of procedure, in studies reporting the risk of bleeding during the periprocedural period, very few or no patients undergoing dental surgery were included. When they were, only low-risk procedures such as extractions were reported and considered (Supplementary Table S1). In a recent review on the perioperative management of patients taking DOACs [44], minor dental procedures (eg, dental extractions, restorations, cleanings, or fillings) were considered as procedures associated with minimal bleeding risk. In addition, most studies do not distinguish between surgical specialties or between the types of procedures within the same specialty. In our study, no significant difference in bleeding risk (total and CRNMB) was observed between high-risk and low-risk oral procedures, underlying the importance of conducting dedicated studies by surgical field to assess the bleeding risk associated with different anticoagulant therapy management strategies.

The present study had several strengths. First, the present study is based on a prospective study including real-world patients on anticoagulants referred for an oral invasive procedure, with anticoagulant treatment management therapy left to the discretion of the dentist. Second, the follow-up comprised the identification and management of both local and general complications during a 30-day follow-up, including 2 visits, one on day 7 as part of the usual follow-up and the other more remotely on day 30. The incidence of TEs among patients in the context of anticoagulant management for oral invasive procedures was not assessed previously in the literature.

Our study was able to include a large number of patients with different anticoagulant therapy management and various oral surgery procedures with different risks of bleeding, enabling us to consider the risks of bleeding in patients receiving DOACs and VKAs, depending on whether they were continued or stopped preoperatively, and to identify risk factors explaining the occurrence of bleeding.

The present study also had several limitations. First, the number of patients was not as high as expected because the funding obtained did not allow recruitment to continue, and the dentists were not paid. Moreover, few patients discontinued oral anticoagulant treatment, leading to wide CIs for the incidence rate of bleeding. No TEs were observed in the study. Therefore, the factors associated with the occurrence of such events could not be investigated. The incidence rate of minor bleeding was lower than expected, which suggests the possibility of underreporting by the patients.

However, despite limited sample size, the study provides important insights regarding the factors associated with hemorrhagic events that occurred during the periprocedural period, with only 3 patients lost to follow-up between days 7 and 30. Second, patients were managed in routine practice, and the study was not randomized. However, there was no difference observed in the characteristics of patients according to their anticoagulant treatment, indications, comorbidities, and sociodemographic traits (except gender) between the patients experiencing bleeds vs the others. The higher risk of bleeding observed in men could not be explained by differences in the proportion of dental procedures at high risk of bleeding between men and women, which was similar (*P* = .85). This result was already reported by Shao et al. [45] and could be explained by suboptimal adherence to postoperative advice, heightened prevalence of smoking habits, or substandard oral hygiene in men [46,47].

In this context, well-conducted studies are needed in this area to properly assess the challenges and the risks in patients on DOACs and to adapt perioperative management in patients undergoing an invasive procedure in general and, more specifically, dental surgery.

Our results are representative of usual management of anticoagulated patients undergoing invasive oral procedures, considering the mode of selection (hospital and private practice dentists with various experience and specialties recruited throughout France).

## 5 Conclusion

Our study reports on the course of oral periprocedural risk of bleeding in anticoagulated patients and illustrates that the risk of bleeding is substantial, occurring mostly during the first 7 days postoperatively. Practitioners and patients should, therefore, be informed of this risk and organize their care so that the patient can be seen again urgently if necessary.

Our results suggest that the periprocedural interruption of DOACs in patients undergoing various invasive oral surgery procedures may be associated with a 30-day periprocedural CRNMB risk of bleeding comparable to that observed with continued VKAs, suggesting the benefit of short perioperative discontinuation of DOACs in this setting to avoid bleeding and emergency referral to the specialist.
