# Supplementary material for: Oral anticoagulant periprocedural management in patients undergoing an oral, dental implant or periodontal surgery: a prospective national observational survey
Source: Res Pract Thromb Haemost. 2025 Apr 10;9(3):102848. doi: 10.1016/j.rpth.2025.102848 (PMC12148435; doi:10.1016/j.rpth.2025.102848)
Supplement: Supplementary Material [file mmc1.docx]

**ORIGINAL ARTICLE**

**ORAL ANTICOAGULANT PERI-PROCEDURAL MANAGEMENT IN PATIENTS UNDERGOING AN ORAL, DENTAL IMPLANT OR PERIODONTALSURGERY: a prospective national observational survey**

**Appendices, Supplementary Material, Supplementary Tables**

**Supplementary Table S1: Dental procedure-related bleeding risk classification**

| **Authors, year** | | **Publication type** | **Procedures with minor/low risk of bleeding** | **Procedures at high risk of bleeding** |
| --- | --- | --- | --- | --- |
| Douketis et al. 2015^1^ | BRIDGE protocol: randomized, double-blind, placebo-controlled trial in patients with AF receiving an elective intervention or procedure; 2 groups: interruption of warfarin therapy and bridging anticoagulation therapy with low-molecular-weight heparin vs placebo | | Dental surgery or other dental procedure without details | Any other surgery or procedure lasting ≥1 hour |
| Syropoulos et al. 2016^2^ | review: patients receiving a vitamin K antagonist or a direct oral anticoagulant requiring an elective procedure or surgery | | Minor dental procedures (dental extractions, restorations, prosthetics, endodontics), dental cleanings, fillings | Any major operation (procedure duration >45 min) |
| Douketis et al. 2019^3^ | PAUSE protocol: prospective study involving DOAC-treated patients with AF who need an elective surgery or procedure (DOAC interruption) 🡪 To investigate the safety of a standardized perioperative DOAC management  strategy | | Dental procedures: - tooth extraction (up to two extractions) - endodontic (root canal) procedure | Other major cancer or reconstructive surgery: head and neck cancer surgery |
| Syropoulos et al. 2019^4^ | review: periprocedural management of patients on chronic oral anticoagulant therapy | | Minor dental procedures (dental extractions, restorations, prosthetics, endodontics), dental cleanings, fillings | Any major operation (procedure duration >45 min) |
| de Andrade et al. 2019^5^ | review: risk of bleeding in patient using oral anticoagulants (most of them VKA): continuation of warfarin vs interruption (risk higher if continuation but not significant) | | Dental minor oral surgical procedure (oral surgery, exodontias) n = 345 - 22 exodontias (simple or several), 2 alveoloplasties and 1 labial frenectomy (Campbel et aL 2000^6^); - 109 exodontias (simple or several) (Evans et al.2002^7^); - 214 exodontias (simple or several) (Al Mubarak et aL 2007)^8^ |  |
| Tafur et al. 2020^9^ | PAUSE protocol: prospective study involving DOAC-treated patients with AF who need an elective surgery or procedure (DOAC interruption) 🡪 To analyze factors associated with perioperative bleedings | | Dental procedures: - tooth extraction (up to two extractions) - endodontic (root canal) procedure | Other major cancer or reconstructive surgery: head and neck cancer surgery |
| Douketis et al. 2024^10^ | Review: perioperative management of patients taking direct oral anticoagulants | | Minor dental procedures (dental extractions, restorations, prosthetics, endodontics), dental cleanings, fillings |  |
| SFCO, 2015^11^ | Recommendations | | Surgeries for which externalized bleeding is easily controlled by conventional surgical hemostasis ● Simple avulsion ● Multiple avulsions in the same quadrant ● Endodontic and periapical surgery (lesion <=2 cm) ● Mucogingival surgery (besides gingival graft with palatine sampling) ● Pre-orthodontic surgery of impacted tooth, included ● Single implant ● Implant(s) release (healing abutment) ●Oral mucosa excisional biopsy (<= 1 cm) | Surgeries with significant blood loss, procedures with operating time > 1-hour, critical procedures by their location (maxillary sinus, floor of the mouth) and/or difficult to control with conventional surgical hemostasis  ● Multiple avulsions in several quadrants ● Dental avulsion(s) of impacted teeth ● Multiple implants in several quadrants ● Sinus lift (crestal approach, lateral approach) ● Apposition bone graft (in onlay) ● Particulate bone grafting and guided bone regeneration ● Surgery and soft tissue (sialolithiasis) ● Enucleation of cysts and benign tumors (lesion > 2 cm) ● Closing an oral sinus communication ● Excision of pseudo tumors and benign tumors of the oral mucosa (> 1 cm) |
|  |  | | **Contraindicated procedures** |  |
|  |  | | ● All procedures contraindicated in case of an associated risk of infective endocarditis |  |
|  |  | | ● All procedures with a risk of bleeding in the case where the technical equipment available to the surgeon is inadequate |  |
|  |  | | ● Bilateral inferior alveolar nerve block: risk of bilateral lateral pharyngeal hematoma and dyspnea |  |
|  |  | | ● Symphyseal sampling: risk of hematoma of the floor of the mouth and dyspnea |  |
|  |  | | ● Autologous graft: not recommended due to an additional collection site, give preference to heterologous and synthetic grafts |  |
|  |  | | ● Gingival graft with palatine sampling: risk of injury to the palatine artery |  |

**Appendices:**

**Investigators and study centers**

| Thong Nguyen | [nguyen.chirplastique@wanadoo.fr](mailto:nguyen.chirplastique@wanadoo.fr) | Université Paris Cité, Hôpital Louis Mourier, service de médecine bucco-dentaire, AP-HP, Colombes, France |
| --- | --- | --- |
| Roch Pecorari | [rochpeco@gmail.com](mailto:rochpeco@gmail.com) | Université Paris Cité, Hôpital Louis Mourier, service de médecine bucco-dentaire, AP-HP, Colombes, France |
| Adeline Loing | [aloing@wanadoo.fr](mailto:aloing@wanadoo.fr) | Université Paris Cité, Hôpital Louis Mourier, service de médecine bucco-dentaire, AP-HP, Colombes, France |
| Jonathan Ravasco | [johnr1988@hotmail.fr](mailto:johnr1988@hotmail.fr) | Université Paris Cité, Hôpital Louis Mourier, service de médecine bucco-dentaire, AP-HP, Colombes, France |
| Tanguy Rouxel | [rouxel.tanguy.91@gmail.com](mailto:rouxel.tanguy.91@gmail.com) | Université Paris Cité, Hôpital Louis Mourier, service de médecine bucco-dentaire, AP-HP, Colombes, France |
| Marion Renoux | [marionrenoux310@yahoo.fr](mailto:marionrenoux310@yahoo.fr) | Université Paris Cité, Hôpital Louis Mourier, service de médecine bucco-dentaire, AP-HP, Colombes ; CH Jacques Monod, Flers, France |
| Anne-Laure Ejeil | [anne-laure.ejeil@wanadoo.fr](mailto:anne-laure.ejeil@wanadoo.fr) | Université Paris Cité, Hôpital Bretonneau, service de médecine bucco-dentaire, AP-HP, Paris, France |
| Nathan Moreau | [nthmoreau@gmail.com](mailto:nthmoreau@gmail.com) | Université Paris Cité, Hôpital Bretonneau, service de médecine bucco-dentaire, AP-HP, Paris, France |
| Marjolaine Gosset | marjolaine.gosset@parisdescartes.fr | Université Paris Cité, Hôpital Charles Foix, département de chirurgie orale, Ivry-sur-Seine, France |
| Nicolas Roche | [dr.nroche@gmail.com](mailto:dr.nroche@gmail.com) | Université Paris Cité, Hôpital Charles Foix, département de chirurgie orale, Ivy-sur-Seine, France |
| Ihsène Taïhi-Nassif | [ihsene.docdoc@gmail.com](mailto:ihsene.docdoc@gmail.com) | Université Paris Cité, Hôpital Rothschild, service de médecine bucco-dentaire, AP-HP, Paris, France |
| Nadia Benlagha | Nadiabenlagha@yahoo.fr | Hôpital André Grégoire, département de chirurgie orale, Montreuil, France |
| Juliette Rochefort | [juliette.rochefort@aphp.fr](mailto:juliette.rochefort@aphp.fr) | Université Paris Cité, Hôpital Pitié-Salpêtrière, service de médecine bucco-dentaire, AP-HP, Paris, France |
| Rafael Toledo Arenas | [rafael.toledo@aphp.fr](mailto:rafael.toledo@aphp.fr) | Université Paris Cité, Hôpital Pitié-Salpêtrière, service de médecine bucco-dentaire, AP-HP, Paris, France |
| Géraldine Lescaille | geraldine.lescaille@gmail.com | Université Paris Cité, Hôpital Pitié-Salpêtrière, service de médecine bucco-dentaire, AP-HP, Paris, France |
| Marc Baranes | [marcbaranes@gmail.com](mailto:marcbaranes@gmail.com) | Cabinet Foch, Saint-Mandé, France |
| Anne-Cécile Becmeur | [anne.cecile.becmeur@gmail.com](mailto:anne.cecile.becmeur@gmail.com) | Cabinet Foch, Saint-Mandé, France |
| Sylvie Boisramé | sylvie.boisrame@chu-brest.fr | UFR d’odontologie, U.B.O, service odontologie médecine bucco-dentaire et chirurgie Orale, CHU Brest, Brest, France |
| Romain Lan | [lanromain@live.fr](mailto:lanromain@live.fr) | Aix-Marseille Université, service de médecine bucco-dentaire, CHU de la Timone, AP-HM, France |
| Jean-Hugues Catherine | [jean-hugues.catherine@ap-hm.fr](mailto:jean-hugues.catherine@ap-hm.fr) | Aix-Marseille Université, service de médecine bucco-dentaire, CHU de la Timone, AP-HM, France |
| Ugo Ordioni | [ugo.ordioni@gmail.com](mailto:ugo.ordioni@gmail.com) | Aix-Marseille Université, service de médecine bucco-dentaire, CHU de la Timone, AP-HM, France |
| Fabrice Campana | [f.campana@centremassiliendelaface.com](mailto:f.campana@centremassiliendelaface.com) | Aix-Marseille Université, service de médecine bucco-dentaire, CHU de la Timone, AP-HM, France |
| Anne-Gaëlle Chaux | [anne-gaelle.chaux@univ-nantes.fr](mailto:anne-gaelle.chaux@univ-nantes.fr) | UFR d'odontologie, Hospices civiles de Lyon, Lyon, France |
| Sylvian Catros | [sylvain.catros@u-bordeaux.fr](mailto:sylvain.catros@u-bordeaux.fr) | Université de Bordeaux, CHU Pellegrin, service de chirurgie orale, Bordeaux, France |
| Jean-Marie Marteau | [jm.marteau33@gmail.com](mailto:jm.marteau33@gmail.com) | Université de Bordeaux, CHU Pellegrin, service de chirurgie orale, Bordeaux, France |
| Johan Samot | [johan.samot@u-bordeaux.fr](mailto:johan.samot@u-bordeaux.fr) | Université de Bordeaux, CHU Pellegrin, service de chirurgie orale, Bordeaux, France |
| Mathilde Fenélon | [mathildefenelon@live.fr](mailto:mathildefenelon@live.fr) | Université de Bordeaux, CHU Pellegrin, service de chirurgie orale, Bordeaux, France |
| Julie Guillet | [julie.guillet@univ-lorraine.fr](mailto:julie.guillet@univ-lorraine.fr) | UFR d’odontologie de Lorraine, CHRU de Nancy Brabois, service de chirurgie orale, Nancy, France |
| Charlene Kichenbrand | [charlene.kichenbrand@gmail.com](mailto:charlene.kichenbrand@gmail.com) | UFR d’odontologie de Lorraine, CHRU de Nancy Brabois, service de chirurgie orale, Nancy, France |
| Bérengère Phulpin | [berengere.phulpin@gmail.com](mailto:berengere.phulpin@gmail.com) | UFR d’odontologie de Lorraine, CHRU de Nancy Brabois, service de chirurgie orale, Nancy, France |
| Nicolas Glock | [dr.nicolasglock@gmail.com](mailto:dr.nicolasglock@gmail.com) | CH St Nicolas, Blaye, France |
| Cécile Chatel | [cchatel1@chu-grenoble.fr](mailto:cchatel1@chu-grenoble.fr) | CHU Grenoble, Grenoble, France |
| Michel Guyot | [drmguyot@gmail.com](mailto:drmguyot@gmail.com) | Cabinet Chirurgie dentaire, pratique privée, Fontenay-le-Comte, France |
| François Allain | [dr.fallain@orange.fr](mailto:dr.fallain@orange.fr) | Cabinet Chirurgie dentaire, pratique privée, Massy, France |
| Julie Bemer | [julie.bemer@ch-havre.fr](mailto:julie.bemer@ch-havre.fr) | GHH, Hôpital Flaubert, Havre, France |
| Alp Alantar | alp.alantar@ch-nanterre.fr | Hôpital Max Fourestier, service de stomatologie, Nanterre, France |
| Catherine Pesci-Bardon | [cat.bardon@orange.fr](mailto:cat.bardon@orange.fr) | Université Côte d’Azur, Faculté de Chirurgie Dentaire Nice, CHU St Roch, Pôle Odontologie, Nice, France |
| Oulimata Diatta-Tricon | [secmeddrtricon@gmail.com](mailto:secmeddrtricon@gmail.com) | Cabinet Chirurgie dentaire, pratique préivée, La Cadiere D'Azur, France |
| Tariq Kabli | [tarik.kabli@live.fr](mailto:tarik.kabli@live.fr) | CHU de Poitiers – Milétrie, service d'odontologie, Poitiers, France |
| Moulay Chemlal | [moulay.chemlal@chu-poitiers.fr](mailto:moulay.chemlal@chu-poitiers.fr) | CHU de Poitiers – Milétrie, service d'odontologie, Poitiers, France |

**Committees**

| **Steering Committee members and affiliations** |
| --- |
| Loredana Radoi, Université Paris Cité, Hopital Louis Mourier, Colombes, France |
| Isabelle Mahé, Université Paris Cité, Hopital Louis Mourier, Colombes, France |
| Louis Maman, SFCO, France |
| Vianney Descroix, Université Paris Cité, Hôpital Pitié-Salpêtrière, Paris, France |
| Virginie Monnet-Corti, SFPIO, France |
| David Hajage, Sorbonne Université, Hôpital Pitié Salpêtrière, Centre de Pharmacoépidémiologie (Cephepi), Paris, France |
| **Statistical Analysis Team** |
| David Hajage, principal statistician, Sorbonne Université, Hôpital Pitié Salpêtrière, Centre de Pharmacoépidémiologie (Cephepi), Paris, France |
| Yann de Rycke, statistician, Sorbonne Université, Hôpital Pitié Salpêtrière, Centre de Pharmacoépidémiologie (Cephepi), Paris, France |
| **CEPHEPI Operational Management of the Study** |
| Nessima Yelles, Centre de Pharmacoépidémiologie (Cephepi), Paris, France |
| **Blinded Adjudication Clinical Events Committee** |
| Loredana Radoi, Université Paris Cité, Hopital Louis Mourier, Colombes, France |
| Isabelle Mahé, Université Paris Cité, Hopital Louis Mourier, Colombes, France |

**Supplementary Material:**

**Endpoint definition**

***Stroke*** was defined as a sudden, focal neurologic deficit resulting from a presumed cerebrovascular cause that is not reversible within 24 hours and not due to a readily identifiable cause, such as a tumor or seizure. Ischemic stroke is defined as an episode of neurological dysfunction caused by focal cerebral, spinal, or retinal infarction^12^.

***Non-cerebrovascular systemic embolism*** was defined as abrupt vascular insufficiency associated with clinical or radiologic evidence of arterial occlusion in the absence of other likely mechanisms (e.g. trauma, atherosclerosis, or instrumentation)^12^.

Diagnosis of ***recurrent VTE*** should be based on objective assessments established by a positive finding on venography, color duplex sonography, ventilation-perfusion lung scanning or spiral computed tomography (CT) Recurrent DVT or PE was diagnosed if the patient had an abnormal compression ultrasound or an intraluminal filling defect on venography or ventilation-perfusion lung scanning or CT in case of documented recanalisation of the initial thrombus, a thrombus in another deep vein in the extremity involved in the previous event, a thrombus in the opposite extremity, or a thrombus in the same vascular system with a proximal extension of the thrombus (if the upper limit of the original thrombus had been visible) or the presence of a constant filling defect surrounded by contrast medium (if the original thrombus had not been visible)^13^.

***Major bleeding*** was defined as clinically overt bleeding associated with any of the following: fatal outcome, involving a critical site (i.e., intracranial, intraspinal, intraocular, pericardial, intraarticular, intramuscular with compartment syndrome, or retroperitoneal), or clinically overt bleeding associated with a fall in hemoglobin concentration of ≥ 2 g/dL, or leading to transfusion of ≥ 2 units of packed red blood cells or whole blood^14^.

***Non-major, clinically relevant bleeding*** is defined as overt bleeding not meeting the criteria for major bleeding but associated with medical intervention, unscheduled contact with a physician (visit or telephone call), temporary (i.e., by delaying the next study drug administration) cessation of study drug, pain, or impairment of daily activities^15^.

All other overt bleeding episodes not meeting the criteria for major or non-major clinically relevant bleeding are classified as ***minor bleeding.***

**Supplementary Table S2: Number of bleedings according to anticoagulant drug (continued or discontinued before the invasive oral procedure)**

|  | **Total population** | **Apixaban**  **N=123** | | **Rivaroxaban**  **N=186** | | **Dabigatran**  **N=36** | | **Total DOAC**  **N=345** | | | **Total VKA**  **N=178** | | |  |  |  |
| --- | --- | --- | --- | --- | --- | --- | --- | --- | --- | --- | --- | --- | --- | --- | --- | --- |
|  | **D+C** | **D** | **C** | **D** | **C** | **D** | **C** | **D** | **C** | **Total** | **D** | **C** | **Total** | **p** | **p*** | **p**** |
|  | **N=523** | **N=10** | **N=113** | **N=20** | **N=166** | **N=2** | **N=34** | **N=32** | **N=313** | **N=345** | **N=9** | **N=169** | **N=178** |  |  |  |
| **Minor Bleedings** |  |  |  |  |  |  |  |  |  |  |  |  |  |  |  |  |
| 24h | 1 | 0 | 0 | 0 | 1 | 0 | 0 | 0 | 1 | 1 | 0 | 0 | 0 | 1.00 | 1.00 | 1.00 |
| Day 7 | 16 | 0 | 5 | 0 | 9 | 0 | 0 | 0 | 14 | 14 | 0 | 2 | 2 | 1.00 | 0.054 | 1.00 |
| Day 30 | 2 | 0 | 0 | 0 | 1 | 0 | 0 | 0 | 1 | 1 | 0 | 1 | 1 | 1.00 | 1.00 | 1.00 |
| **Total** | 19 | 0 | 5 | 0 | 11 | 0 | 0 | 0 | 16 | 16 | 0 | 3 | 3 | - | - | - |
| At least 1 event within 30 Days | 19 | 0 | 5 | 0 | 11 | 0 | 0 | 0 | 16 | 16 | 0 | 3 | 3 | 1 | 0.072 | 1.000 |
| **Incidence**  **per 100 PM** | **3.63** | **0.00** | **4.42** | **0.00** | **6.63** | **0.00** | **0.00** | **0.00** | **5.11** | **4.64** | **0.00** | **1.78** | **1.69** | 1.00 | 0.078 | 0.972 |
| **CRNMB** |  |  |  |  |  |  |  |  |  |  |  |  |  |  |  |  |
| 24h | 18 | 0 | 7 | 1 | 8 | 0 | 0 | 1 | 15 | 16 | 0 | 2 | 2 | 1.00 | **0.040** | 0.407 |
| Day 7 | 21 | 1 | 8 | 0 | 8 | 0 | 0 | 1 | 16 | 17 | 0 | 4 | 4 | 1.00 | 0.149 | 0.584 |
| Day 30 | 4 | 0 | 0 | 0 | 3 | 0 | 0 | 0 | 3 | 3 | 0 | 1 | 1 | 1.00 | 1.00 | 1.00 |
| **Total** | 43 | 1 | 15 | 1 | 19 | 0 | 0 | 2 | 34 | 36 | 0 | 7 | 7 | - | - | - |
| At least 1 event within 30 Days | 36 | 1 | 13 | 1 | 15 | 0 | 0 | 2 | 28 | 30 | 0 | 6 | 6 | 1.00 | **0.027** | 0.616 |
| **Incidence**  **per 100 PM** | **8.22** | **10.00** | **13.27** | **5.00** | **11.45** | **0.00** | **0.00** | **6.25** | **10.86** | **10.43** | **0.00** | **4.14** | **3.93** | 0.917 | **0.016** | 0.951 |
| **Local Bleedings** |  |  |  |  |  |  |  |  |  |  |  |  |  |  |  |  |
| 24h | 19 | 0 | 7 | 1 | 9 | 0 | 0 | 1 | 16 | 17 | 0 | 2 | 2 | 1.00 | **0.030** | 0.407 |
| Day 7 | 37 | 1 | 13 | 0 | 17 | 0 | 0 | 1 | 30 | 31 | 0 | 6 | 6 | 1.00 | **0.016** | 1.00 |
| Day 30 | 6 | 0 | 0 | 0 | 4 | 0 | 0 | 0 | 4 | 4 | 0 | 2 | 2 | 1.00 | 1.00 | 1.00 |
| **Total** | 62 | 1 | 20 | 1 | 30 | 0 | 0 | 2 | 50 | 52/345 | 0 | 10 | 10 | - | - | - |
| At least 1 event within 30 Days | 50 | 1 | 16 | 1 | 24 | 0 | 0 | 2 | 40 | 42 | 0 | 8 | 8 | 1.00 | **0.005** | 0.662 |
| **Incidence**  **per 100 PM** | **11.85** | **10.00** | **17.70** | **5.00** | **18.07** | **0.00** | **0.00** | **6.25** | **15.97** | **15.07** | **0.00** | **5.92** | **5.62** | 0.917 | **0.003** | 0.746 |

D : Discontinued ; C : Continued

P : Comparison of the bleedings in patients with DOAC discontinuation vs those with VKA discontinuation

P* : Comparison of the bleedings in patients with DOAC continuation vs those with VKA continuation

P** : Comparison of the bleedings in patients with DOAC discontinuation vs VKA continuation

**References**

1. Douketis JD, Spyropoulos AC, Kaatz S, et al. Perioperative Bridging Anticoagulation in Patients with Atrial Fibrillation. *N Engl J Med*. 2015;373(9):823-833. doi:10.1056/NEJMoa1501035

2. Spyropoulos AC, Al-Badri A, Sherwood MW, Douketis JD. Periprocedural management of patients receiving a vitamin K antagonist or a direct oral anticoagulant requiring an elective procedure or surgery. *J Thromb Haemost*. 2016;14(5):875-885. doi:10.1111/jth.13305

3. Douketis JD, Spyropoulos AC, Duncan J, et al. Perioperative Management of Patients With Atrial Fibrillation Receiving a Direct Oral Anticoagulant. *JAMA Intern Med*. 2019;179(11):1469-1478. doi:10.1001/jamainternmed.2019.2431

4. Spyropoulos AC, Brohi K, Caprini J, et al. Scientific and Standardization Committee Communication: Guidance document on the periprocedural management of patients on chronic oral anticoagulant therapy: Recommendations for standardized reporting of procedural/surgical bleed risk and patient‐specific thromboembolic risk. *Journal of Thrombosis and Haemostasis*. 2019;17(11):1966-1972. doi:10.1111/jth.14598

5. de Andrade NK, Motta RHL, Bergamaschi C de C, et al. Bleeding Risk in Patients Using Oral Anticoagulants Undergoing Surgical Procedures in Dentistry: A Systematic Review and Meta-Analysis. *Front Pharmacol*. 2019;10:866. doi:10.3389/fphar.2019.00866

6. Campbell JH, Alvarado F, Murray RA. Anticoagulation and minor oral surgery: should the anticoagulation regimen be altered? *J Oral Maxillofac Surg*. 2000;58(2):131-135; discussion 135-136. doi:10.1016/s0278-2391(00)90324-0

7. Evans IL, Sayers MS, Gibbons AJ, Price G, Snooks H, Sugar AW. Can warfarin be continued during dental extraction? Results of a randomized controlled trial. *Br J Oral Maxillofac Surg*. 2002;40(3):248-252. doi:10.1054/bjom.2001.0773

8. Al-Mubarak S, Al-Ali N, Abou-Rass M, et al. Evaluation of dental extractions, suturing and INR on postoperative bleeding of patients maintained on oral anticoagulant therapy. *Br Dent J*. 2007;203(7):E15; discussion 410-411. doi:10.1038/bdj.2007.725

9. Tafur AJ, Clark NP, Spyropoulos AC, et al. Predictors of Bleeding in the Perioperative Anticoagulant Use for Surgery Evaluation Study. *J Am Heart Assoc*. 2020;9(19):e017316. doi:10.1161/JAHA.120.017316

10. Douketis JD, Spyropoulos AC. Perioperative Management of Patients Taking Direct Oral Anticoagulants: A Review. *JAMA*. 2024;332(10):825-834. doi:10.1001/jama.2024.12708

11. Gestion péri-opératoire des patients traités par antithrombotiques en chirurgie orale. Recommandations. *Med Buccale Chir Buccale*. 2015;21:S5-S14. doi:10.1051/mbcb/2015036

12. Sacco RL, Kasner SE, Broderick JP, et al. An Updated Definition of Stroke for the 21st Century: A Statement for Healthcare Professionals From the American Heart Association/American Stroke Association. *Stroke*. 2013;44(7):2064-2089. doi:10.1161/STR.0b013e318296aeca

13. Eichinger S, Heinze G, Jandeck LM, Kyrle PA. Risk Assessment of Recurrence in Patients With Unprovoked Deep Vein Thrombosis or Pulmonary Embolism: The Vienna Prediction Model. *Circulation*. 2010;121(14):1630-1636. doi:10.1161/CIRCULATIONAHA.109.925214

14. Schulman S, Kearon C, Subcommittee on Control of Anticoagulation of the Scientific and Standardization Committee of the International Society on Thrombosis and Haemostasis. Definition of major bleeding in clinical investigations of antihemostatic medicinal products in non-surgical patients. *J Thromb Haemost*. 2005;3(4):692-694. doi:10.1111/j.1538-7836.2005.01204.x

15. Kaatz S, Ahmad D, Spyropoulos AC, Schulman S, Subcommittee on Control of Anticoagulation. Definition of clinically relevant non-major bleeding in studies of anticoagulants in atrial fibrillation and venous thromboembolic disease in non-surgical patients: communication from the SSC of the ISTH. *J Thromb Haemost*. 2015;13(11):2119-2126. doi:10.1111/jth.13140
